# Psychological Burden and Associated Factors of the COVID-19 Pandemic on People in Quarantine and Isolation Centers in Ethiopia: A Cross-Sectional Study

**DOI:** 10.3389/fpsyt.2021.753383

**Published:** 2022-01-12

**Authors:** Tadesse Misgana, Dejene Tesfaye, Mandaras Tariku, Tilahun Ali, Daniel Alemu, Yadeta Dessie

**Affiliations:** ^1^Department of Psychiatry, College of Health and Medical Sciences, Haramaya University, Harar, Ethiopia; ^2^School of Public Health, College of Health and Medical Sciences, Haramaya University, Harar, Ethiopia

**Keywords:** suspected cases, quarantine, isolation, common mental disorder, COVID-19, Ethiopia

## Abstract

**Background:** Globally, a lot of countries put into practice early quarantine measures as an essential COVID-19 prevention mechanism. Other than physical effects, quarantine has a major result on mental health and well-being at both the individual as well as the community level at large. Therefore, this study aimed to assess the psychological burden of COVID-19 on the people in quarantine and isolation centers and to identify associated factors for early and effective psychosocial intervention during the pandemic and beyond.

**Method:** A cross-sectional study was done among 392 suspected cases of COVID-19 that were in quarantine and isolation centers found in Eastern Ethiopia in 2020. Participants were selected by the convenience sampling method. The common mental disorder was measured by the Self Reporting Questionnaire-20 (SRQ-20). Logistic regression was done to identify predictive factors, and a *P* < 0.05 was considered statistically significant.

**Results:** The common mental disorder among suspected cases of COVID-19 in Ethiopia was found to be 13.5% (95% CI: 10.2, 17.1%). Female (AOR = 1.52, 95% CI: 1.1, 2.92), known chronic medical illness (AOR = 7.0, 95% CI: 2.2, 21.8), inadequate accessibility of personal protective equipment (AOR = 6.1, 95% CI: 2.8, 13.3), poor awareness about the pandemic (AOR = 2.90, 95% CI: 2.71, 7.54), presence of symptoms of the disease (AOR = 5.3, 95% CI: 2.57, 11.1), and substance use (AOR = 2.7, 95% CI: 1.2, 6.1) were found to be associated with a common mental disorder.

**Conclusion:** The current study revealed that the common mental disorder was relatively high among suspected cases of COVID-19 in quarantine and isolation centers as compared with the general population. The results of the present study demonstrate that some subpopulations are more vulnerable to the pandemic's deleterious effects on mental health. Therefore, providing appropriate psychosocial intervention for the populations at risk is important to decrease the effect of common mental disorders among suspected cases of COVID-19.

## Introduction

The novel coronavirus disease (COVID-19) was identified for the first time in Wuhan, China, in December 2019 (1), and the WHO affirmed it as a worldwide pandemic on March 11, 2020 (2). It is accompanied by respiratory conditions such as respiratory failure, respiratory distress syndrome, and septic shock. In most cases, patients with COVID-19 may improve without extraordinary management although those with advanced age and preexisting physical illnesses are highly prone to have severe symptoms and illness and may progress to death (1, 3).

Quarantine and isolation became increasingly applied around the world to contain the transmission of the virus (4). Besides practicing different COVID-19 control measures, such as hand washing or sanitizing, use of face masks, and physical distancing, most countries are demanding to provide economic assistance to several people who lost their jobs due to lockdown and closure of nonessential commercial activity (5, 6). Despite those control measures, in the framework of serious threats to everyone's health and livelihood with the irregularity of governance efforts, extensive mental health, substance use, and psychosocial problems seem estimated to occur, both directly from the pandemic and indirectly from the related economic recession. It is known that such a pandemic has a major impact on mental health, and this problem has been described as a “parallel epidemic” (7).

There are various reasons for the impact of the pandemic on mental health. People now are suddenly facing major changes in their daily life, working models, and social behaviors. It is not surprising that several individuals are showing acute fight-or-flight responses, such as increased anxiety levels, panic attacks, irrational fears up to paranoid-like convictions, and related behaviors (8, 9). Some are experiencing losses under traumatic circumstances, such as not being able to say goodbye to dying loved ones or the inability to offer proper burials (10, 11). The disease itself is multiplied as forced mass quarantine to battle COVID-19 can cause depression, anxiety, and distress (12) due to factors such as the sense of getting worried and loss of control (13). This can be severe if families need separation by the uncertainty of disease status, scarcity of essentials, economical constraints, and increased susceptibility to the disease, which usually get exaggerated by unclear information and inappropriate communications via media (5, 14, 15). “The potential benefits of mandatory mass quarantine need to be weighed carefully against the possible psychological costs as studies inform different mental health conditions that may appear when an individual is quarantined or isolated” (5).

Even though the WHO has strategically announced relevant policies and principles, the COVID-19 pandemic has caused a grave challenge on mental health services particularly among suspected and confirmed cases and frontline health care workers. They may develop a fear of contagion, which as a result, may lead to suicidal behaviors, sleep disturbances, and stress-related disorders. Further, firm quarantine and mandatory contact tracing policies by health authorities could cause societal rejection, discrimination, and stigmatization (5).

Previous studies conducted worldwide about the impact of COVID-19 on mental health show that symptoms of depression, anxiety, and unexplained somatic symptoms are common emotional and psychological responses to the pandemic (16, 17). The communities were concerned about social isolation accompanied by quarantine during the pandemic (18). According to a study done in China, which determines the impact of the COVID-19 pandemic, the majority of the study respondents reported a different level of symptoms of common mental disorders. In this study, about 16% of the participants reported different levels of depression, 35% reported different symptoms of anxiety, and 8% reported moderate-to-severe distress (19).

Developing countries traditionally have a higher prevalence of mental disorders (20, 21). Therefore, it is reasonable to assume that, in the pandemic, this situation worsened. Although the tremendous psychological impact of the pandemic is widely discussed, a burden of the psychological impact of this pandemic on the peoples in quarantine and isolation centers is not yet well studied in developing countries, including Ethiopia. It is very significant to study the mental health status of the people in quarantine to develop and implement effective interventions to mitigate the impact of psychological disorders in this grave pandemic era. Therefore, this study was intended to assess the psychological burden of COVID-19 on the people in quarantine and isolation centers and to identify associated factors for early and effective psychosocial intervention during the pandemic and beyond.

### Hypothesis

Ho_1_: There is no psychological impact of COVID-19 among suspected cases in quarantine and isolation centers.
Ho_2_: All suspected cases of COVID-19 with different characteristics are equally experiencing psychological illnesses.

## Materials and Methods

### Study Period, Area, and Design

A cross-sectional study was done in Harari and Oromia Regional state, which is found in Eastern Ethiopia, from November 15 to December 31, 2020. During this period, the pandemic was highly contagious, the numbers of cases were dramatically increased in Ethiopia, and this would cause a greater mental disorder.

Harari Regional state is divided into six urban and three rural districts. According to the 2007 national population census, the total population of the region was 183,344, of which 99,321 (54.2 %) were urban and 84,023 (45.8 %) were rural residents. There are two public and one police hospital in region. The East Hararghe zone of Oromia regional state is one of the most populous zones of the region with more than 2.7 million people. It is divided into 18 districts and has three public hospitals (22).

### Characteristics of Participants

All suspected cases of COVID-19 in people aged 18 years and above and who were in quarantine and isolation centers at Harari regional state and East Hararghe Zones, Eastern Ethiopia, during the data collection period were included in the study. Suspected cases of people with COVID-19 who cannot communicate due to severe health conditions were excluded from the study.

### Sampling Technique

A convenience sampling method was applied to select the respondents. A total of 392 suspected cases who were identified by the rapid response team of the Harari regional state health bureau and eastern Hararghe zonal health department and available in quarantine and isolation centers during the study period were interviewed face to face (direct contacts), considering all the necessary precautions recommended by WHO that were kept to prevent the transmission of COVID-19.

### Data Collection Instruments

The common mental disorder was assessed by using the Self-Reporting Questionnaire (SRQ-20). It contains 20 “yes” or “no” questions that assess the symptoms of depression, anxiety, and unexplained somatic complaints in the previous 30 days (23). The SRQ-20 has been tested in various settings with different cutoff points. The SRQ-20 was validated in Ethiopia at a general population, and a cutoff point of six was found to have a sensitivity of 90.7% and specificity of 80.7% (24). For this study, the cutoff point six was considered as the presence of common mental disorders, and the reliability in this study was kappa value of 0.89. People scoring above six were linked and underwent a full clinical psychiatric examination by licensed psychiatrists.

Semistructured questionnaires were applied to review the demographic, clinical, and COVID-19-related descriptions of the participants. Sociodemographic and economic characteristics include age, sex, educational status, religion, occupation, marital status, and residence. Clinical variables include previous history of mental illness and known preexisting chronic medical illness.

COVID-19 control measures, knowledge about COVID-19, time spent focusing on COVID-19 information, presence of adequate personal protective equipment, any symptoms of COVID-19, and perceived severity of COVID-19 were included in the study as COVID-19-related characteristics. Participants' knowledge of COVID-19 was assessed based on responses to the following COVID-19-related single-topic questions: 1) “What kind of information have you received about COVID 19?” with possible response options being how to protect yourself from the disease, symptoms of COVID-19, how it is transmitted, what to do if they have the symptoms, and risks and complications of illness; 2) “what are the major symptoms of COVID 19?” with possible response options being cough, shortness of breath, sore throat, runny or stuffy nose, muscle or body aches, headaches, fatigue (tiredness), diarrhea, and loss of taste and smell; and 3) “how do you rate your level of knowledge on how to prevent the spread of COVID-19 (face mask, hand washing/sanitizing, physical distancing or other)?” with possible response options being little, very poor, poor, good, and very good.

Substance use behavior of the participants was assessed by the Alcohol, Smoking and Substance Involvement Screening Test (ASSIST). It is a brief, standardized WHO screening questionnaire to find out about participant's use of psychoactive substances with a sensitivity and specificity of 97 and 90%, respectively. It comprises eight questions or items, covering 10 substances: tobacco, alcohol, cannabis, cocaine, amphetamine-type stimulants (ATS), inhalants, sedatives, hallucinogens, opioids, and “other” drugs. For the purpose of this study, *Khat* was included under the stimulant category. The ASSIST also investigates frequency of use and associated problems for each substance (25).

Social support of the study participants was assessed by the Oslo 3-item Social Support Scale (OSSS-3). It is a brief and economic instrument for the assessment of the level of social support (26). In a Nigerian population, the internal consistency of the OSSS-3 was accepted with a Cronbach's alpha coefficient of 0.50 and the concurrent validity of OSSS-3 with the depression subscale of the Hospital Anxiety Depression Scale (HADS) was low but significant and inversely related. The discriminate validity was good and was shown by the significant difference between mean OSS-3 of HADS-depression subscale cases compared with non-cases (*t*-test = 6.710; *p* ≤ 0.0001; *r* = −0.3; *p* = 0.011) (27).

The OSSS-3 contains three items assessing the number of close confidantes, perceived level of concern from others, and perceived ease of getting help from neighbors. The OSSS-3 sum score can be operationalized into three broad categories of social support; 3–8 poor social support, 9–11 moderate social support, and 12–14 strong social support (28).

### Data Collection Procedures

Data were collected from suspected COVID-19 individuals using face-to-face interviews by 25 trained health professionals (nurses) working under the districts' (woredas') rapid response team of the Harari region health bureau region and eastern Hararghe zonal health department. Four supervisors were monitoring the data collection process at each site. During the data collection period, all the necessary precautions recommended by WHO were kept to prevent the transmission of COVID-19.

### Statistical Analysis

EpiData (version 3.1, EpiData Association, Odense, Denmark) was used to enter the cleaned data, and STATA (version 14.0, StataCorp LLC, College Station, Texas, USA) was used for analyzing the data. Categorical variables were described using frequency and percentage, and continuous variables were described using mean and standard deviation (SD). Explanatory factors that have been associated with a common mental disorder were identified by conducting bivariable and multivariable logistic regression, and a *p* < 0.05 was considered statistically significant. For all of the models, variance inflation factor (VIF) was used to test multicollinearity of independent variables, and the Hosmer and Lemeshow test was done to check model adequacy.

### Ethical Consideration

Ethical clearance was obtained from the Institutional Health Research Ethics Review Committee (IHRERC) of the College of Health and Medical Sciences, Haramaya University, with the approval number of IHRERC/243/2020. The permission letter was submitted to Harari regional state and east Hararghe zone COVID-19 task force office. Before the questionnaire was administered to any eligible participant, informed, voluntary, written and signed consent was obtained from each participant.

## Results

### Sociodemographic Descriptions of Study Participants

Of 423 suspected cases selected for the study, 392 consented to participate, which yields a response rate of 92.7%. More than half of the respondents, 244 (62.2%) were less than 30 years old with a mean age of 28.9 years (±11.2). A total of 187 (47.7%) participants were females. Orthodox religion followers accounted for 145 (37%), and 172 (43.9%) were Oromo in ethnicity. The majority of the study participants, 204 (52%), were married, and 100 (25.5%) were government employees. Around 115 (29.3%) of the participants were joining the college for the learning, and 346 (88.3%) were living in urban areas as shown in Table 1.

**Table 1:** Sociodemographic characteristics of suspected cases in quarantine and isolation centers due to COVID-19 in Eastern Ethiopia, 2020.

**Variables**	**Category**	**Frequency (***n*** = 392)**	**Percentage (%)**
Sex	Male	205	52.3
	Female	187	47.7
Age (years)	<30	244	62.2
	30–39	81	20.7
	40–49	47	12.0
	>50	20	5.1
Marital status	Never married	179	45.7
	Married	204	52.0
	Others*	9	2.3
Religion	Orthodox	145	37.0
	Protestant	84	21.4
	Muslim	140	35.7
	Others***	23	5.9
Ethnicity	Oromo	172	43.9
	Amhara	111	28.3
	Harari	21	5.4
	Tigre	25	6.4
	Gurage	48	12.2
	Others**	15	3.8
Educational status	Cannot write and read	39	9.9
	Only able to write and read	74	18.9
	Primary school	63	16.1
	Secondary and preparatory school	101	25.8
	College and above	115	29.3
Occupational status	Jobless	39	9.9
	Merchant	80	20.4
	Farmer	24	6.1
	Student	104	26.5
	Government employee	100	25.5
	Housewife	36	9.2
	Others	9	2.3
Residence	Urban	346	88.3
	Rural	46	11.7

Others*= divorced, separated, widowed;

Others**= Wolayita, Silte;

Others***= NGO, daily laborer. Others*** = Catholic, indigenous religions.

### COVID-19 Related and Clinical Descriptions

Of the respondents, eight (2.0%) had a preexisting mental disorder, and 25 (6.4%) had a chronic medical illness. About 74 (18.9%) of the study participants didn't practice any types of COVID-19 control measures. Of the study participants practicing control measures of COVID-19, hand washing or sanitizing 260 (66.3%) were the most common followed by proper use of face masks 159 (40.7%). About 101 (25.8%) of the participants have no adequate accessibility to personal protective equipment (26), and 205 (52.3%) were seeking information about COVID-19 two times daily. Regarding the knowledge of COVID-19, 227 (57.9%) of them received information on how to protect themselves from the disease, whereas 193 (49.2%) of them received information on how it is transmitted. The majority, 262 (66.8%), of the participants perceived that they have good knowledge about the COVID-19 pandemic (Table 2).

**Table 2:** Clinical and COVID-19 related characteristics of suspected cases in quarantine and isolation centers due to COVID-19 in Eastern Ethiopia, 2020.

**Variables**	**Category**	**Frequency (***n*** = 392)**	**Percentage (%)**
History of mental illness	Yes	8	2.0
	No	384	98.0
History of chronic medical illness	Yes	25	6.4
	No	367	93.6
Practicing COVID-19 control measures	Yes	74	18.9
	No	318	81.1
Accessibility of adequate Personal protective equipment	Yes	291	74.2
	No	101	25.8
Time spent focusing on COVID-19 information	None	71	18.1
	Two times daily	205	52.3
	Three and more times daily	116	29.6
Symptoms of COVID-19	Yes	106	27.0
	No	286	73.0
Self-rated severity of the COVID-19	Mild	80	74.1
	Moderate	26	24.1
	Severe	2	1.9
Kind of information received about COVID-19	How to protect from the disease	227	57.9
	Symptoms of COVID-19	312	79.6
	Modes of transmission	193	49.2
	What to do if they have the symptoms	72	18.4
	Risk and complications of illness	94	24.0
Perceived knowledge about COVID-19	Poor	130	33.2
	Good	262	66.8

**Figure 1:**
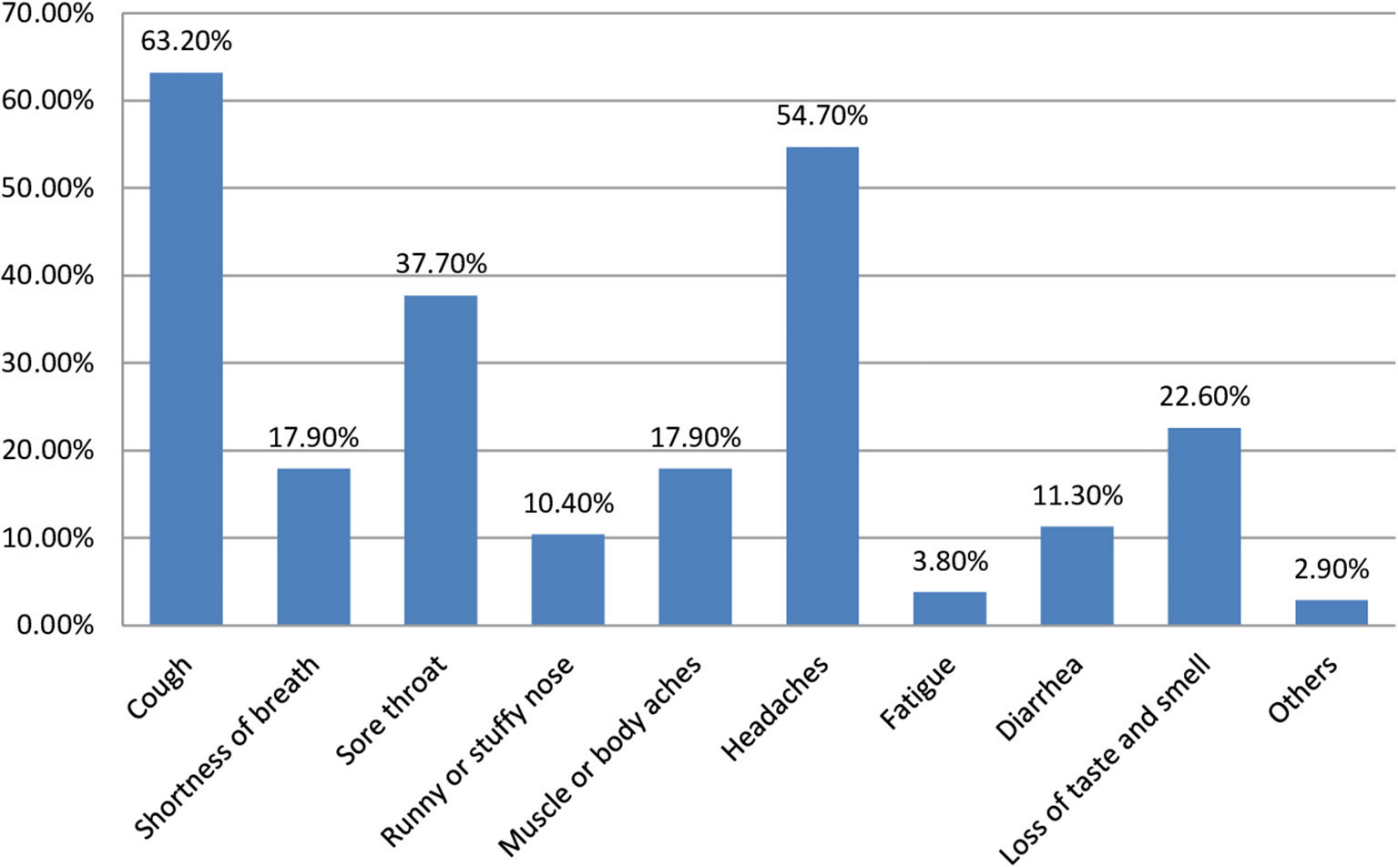
Description of signs and symptoms manifested by the suspected cases in quarantine and isolation centers due to COVID-19 in Eastern Ethiopia, 2020.

About 106 (27%) of the study participants had symptoms of COVID-19 pandemic, and of this, cough 67 (63.2%) is the most prevalent symptom; 80 (74.1%) of them rated their symptoms as mild (Figure 1).

### Substance Use Behavior and Psychosocial Related Characteristics of the Participants

**Figure 2:**
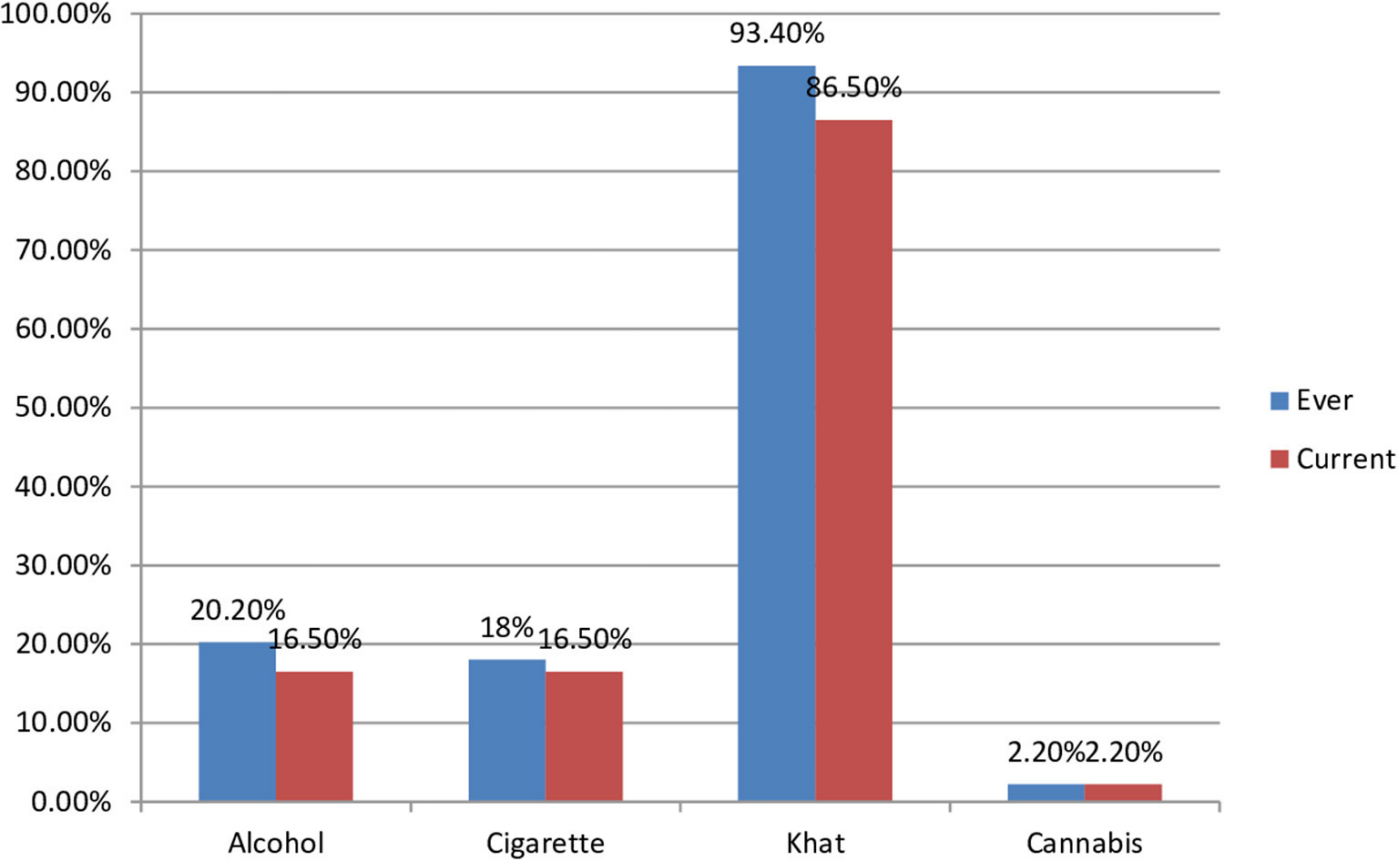
Description of types of substance used by he suspected cases in quarantine and isolation centers due to COVID-19 in Eastern Ethiopia, 2020.

The substance use behavior of the participants was assessed using ASSIST. Accordingly, 91 (23.2%) were using a substance at least once throughout their lifetime, and 89 (22.7%) were using at least one type of substance within the last 3 months as shown in Figure 2. Regarding receiving the support, 165 (42.1%) of the study participants had good social support, and 57 (14.5%) had poor social support.

### Prevalence of the Common Mental Disorder

The overall prevalence of a common mental disorder among suspected cases in quarantine and isolation centers due to COVID-19 was found to be 13.5% [95% CI: 10.2, 17.1%]. Concerning the distribution of the symptoms, headache (*n* = 66) was the symptom experienced most frequently, followed by having a poor appetite (*n* = 48). The mean of SRQ-20 score was 3 (SD = 1).

### Factors Associated With a Common Mental Disorder

In the bivariable logistic regression model: age, sex, marital status, educational status, residence, chronic medical illness, presence of adequate personal protective equipment, perceived knowledge about COVID-19, presence of any symptoms of COVID-19, and current use of any substance were associated with a common mental disorder and considered for multivariable analysis.

The final multivariable logistic regression model revealed the following: Female sex (AOR = 1.52, 95% CI: 1.1, 2.92), known chronic medical illness (AOR = 7.0, 95% CI: 2.2, 21.8), inadequate accessibility of personal protective equipment (AOR = 6.1, 95% CI: 2.8, 13.3), poor awareness concerning the pandemic (AOR = 2.90, 95% CI: 2.71, 7.54), presence of symptoms of the disease (AOR = 5.3, 95% CI: 2.57, 11.1), and substance use (AOR = 2.7, 95% CI: 1.2, 6.1) were found to be associated with a common mental disorder (Table 3).

**Table 3:** Factors associated with common mental disorder among suspected cases in quarantine and isolation centers due to COVID-19 in Eastern Ethiopia, 2020.

**Explanatory variable**	**Category**	**Common mental disorder**		**COR (95% CI)**	**AOR (95% CI)**	***P*** **-value**
		**Yes**	**No**			
Age (years)	<30	21	223	1	1	
	30–39	14	67	2.22 (1.07, 4.60)	2.06 (0.74, 5.73)	0.17
	40–49	11	36	3.25 (1.44, 7.29)	1.67 (0.53, 5.25)	0.38
	>50	7	13	5.72 (2.06, 15.89)	3.44 (0.67, 17.5)	0.14
Sex	Female	32	155	1.81 (1.01, 3.27)	1.52 (1.1, 2.92)	0.04
	Male	21	184	1	1	
Marital status	Never married	15	164	1	1	
	Married	33	171	2.11(1.11, 4.03)	1.53 (0.59, 3.96)	0.38
	Divorced/widowed	5	4	13.67 (3.31, 56.38)	5.79 (0.83, 40.4)	0.77
Residence	Urban	44	302	1	1	
	Rural	9	37	1.67 (0.76, 3.69)	1.47 (0.52, 4.12)	0.46
Educational status	Unable to write and read	9	30	3.15 (1.17, 8.46)	1.69 (0.49, 5.76)	0.40
	Able to write and read	16	58	2.90 (1.24, 6.79)	1.27 (0.41, 3.89)	0.67
	Primary school	7	56	1.31 (0.47, 3.64)	1.02 (0.31, 3.44)	0.97
	Secondary/preparatory school	11	90	1.28 (0.52, 3.16)	1.99 (0.68, 5.81)	0.20
	College and above	10	105	1	1	
Chronic medical illness	Yes	9	16	4.13 (1.72, 9.91)	7.0 (2.2, 21.8)	0.001
	No	44	323	1	1	
Presence of adequate personal protective equipment	Yes	26	265	1	1	
	No	27	74	3.72 (2.05, 6.76)	6.1 (2.8, 13.3)	<0.001
Any symptoms of COVID-19	Yes	33	73	6.01 (3.26, 11.10)	5.3 (2.57, 11.1)	<0.001
	No	20	266	1.00	1.00	
Knowledge about COVID-19	Poor	30	100	3.1 (2.84, 6.37)	2.90 (2.71, 7.54)	0.02
	Good	23	239	1	1	
Current use of any substance	Yes	23	66	3.17 (1.73, 5.8)	2.7 (1.2, 6.1)	0.014
	No	30	273	1	1	

*AOR, adjusted odds ratio; COR, Crude Odds Ratio; CI, Confidence Interval; COVID-19, Corona Virus Disease 2019*.

## Discussion

The present study utilizing a sample of 392 Ethiopian adults found that the common mental disorder was 13.5% in suspected cases of COVID-19 who were self-isolating and in a quarantine center due to the COVID-19 pandemic. The finding was comparable with studies done in China 11.9% (29), but it was lower as compared with the studies done in the United States 69% (30), China 36.05% (31), and Canada, which revealed the prevalence of depression and anxiety as 31.2 and 28.9%, respectively (14, 32). The possible explanation for this difference might be due to the government introducing the rapid response team to prevent and control the disease, which includes a psychological first aid team. Besides this, this study was conducted in December 2020 when the incidence of the cases decreased, indicating the quick transmission of the virus had been controlled, which alleviated psychological distress and symptoms of common mental disorder symptoms. On the other hand, the result was higher than the study conducted in another part of China at 9.0% (33). This discrepancy might be due to the variation in demographic characteristics, sampling size, and data collection instrument used to measure the dependent variable.

After adjusting for the potential confounder, being female, having known chronic medical illness, having inadequate accessibility of personal protective equipment, having poor knowledge about the COVID-19 pandemic, complaining of any symptoms of COVID-19, and currently using any type of substances were associated with a common mental disorder.

In this study, female sex was significantly associated with the common mental disorder. This result was consistent with studies conducted in Spain (34), the United Kingdom (35), and China (31). The possible explanation for this might be due to the affective nature of a female's response to stressful life events and hormonal changes. Second, during the virus pandemic, different studies reported the increments in domestic violence, and home quarantine may exacerbate gender-based violence (36).

Suspected cases of COVID-19 who had any type of chronic disease were seven times more likely to have a common mental disorder. This result agrees with a study done in the United Kingdom (37). In this situation, patients with chronic illnesses, cardiovascular diseases, cancer, diabetes, hypertension, stroke, cognitive disorders, and psychotic disorders experience emotional disturbance, nervousness, rage, perplexity, and stigma (5, 38) due to unexpected separation from loved ones, scarcity of essentials, the loss of freedom, and uncertainty over disease status. In addition, some patients have been confronted with difficulties in regular medical supplies due to delayed transportation and scarcity of medicines and human power in health facilities (39). Moreover, in this pandemic, the death rate in aged patients with chronic illness is the highest. Some older patients dared not go to the hospital. All these circumstances raise the possibility of relapse or even death.

According to this study, inadequate accessibility of personal protective equipment was significantly associated with the common mental disorder. This finding agreed with a study done in the United States (40). “Personal protective equipment is recommended in the care of anyone with suspected COVID-19 by multiple health organizations including the World Health Organization and the Centers for Disease Control and Prevention, forcing physicians to choose between protecting themselves and caring for their patients” (41). This causes specific sources of worry and panic among frontline health care worker that are combating the COVID-19 outbreak.

The other important predictor of a common mental disorder was having the common signs and symptoms of the COVID-19 disease. This result was in accordance with studies done in Spain (34) and Wuhan, China (42). In the current study, people with suspected cases of COVID-19 who had low awareness of the pandemic were 2.4 times more likely to have common mental disorders. The finding was consistent with the study done in Spain (34). The role of information seems to be fundamental and influences the mental health status of the individuals.

It is also worth noting that currently using any type of substance was significantly associated with the development of a common mental disorder (AOR = 2.7, 95% CI: 1.2, 6.1). This result was supported by studies conducted in the United Kingdom (43) and the United States (44). There are several possible explanations for this result. Existing literature shows that exposure to a traumatic event is particularly linked to starting substance use and that using the substance in response to stress has been specifically linked to an increased possibility of developing a substance use disorder and other common mental illness (45).

The finding of this study support the notion that public mental health interventions should be formally integrated into public health preparedness and emergency response plans. Mental health-care organizations and public health institutions are releasing practical guidelines on taking care of mental health and well-being (46, 47). The American Psychiatric Association (APA), the National Alliance on Mental Illness (NAMI), and the Substance Abuse and Mental Health Services Administration (SAMHSA) provide general tips for the community on how to organize their own time and manage their physical and mental health. The Centers for Disease Control and Prevention and the WHO supply further information specific for high-risk groups (48, 49).

## Limitation of the Study

The study has some limitations. Considering the limited availability of resources and the urgent or alarming effect of the COVID-19 pandemic outbreak, we implemented the convenience sampling technique. This sampling strategy was not based on a random selection of the sample, and the study population did not reveal the actual pattern of the general population.

## Conclusion

The current study revealed that having a common mental disorder was relatively high among suspected cases of COVID-19 as compared with the general populations. Being female, having known chronic medical illness, inadequate accessibility of personal protective equipment, having poor knowledge about the COVID-19 pandemic, complaining of any symptoms of COVID-19, and currently using any type of substance was associated with a common mental disorder. Therefore, providing appropriate psychosocial intervention for the populations at risk is important to decrease the effect of common mental disorders among suspected cases of COVID-19.

## Data Availability Statement

The raw data supporting the conclusions of this article will be made available by the authors, without undue reservation.

## Ethics Statement

The studies involving human participants were reviewed and approved by Institutional Health Research Ethics Review Committee (IHRERC) of the College of Health and Medical Sciences, Haramaya University. The patients/participants provided their written informed consent to participate in this study.

## Author Contributions

All authors were contributed to the inception of the study, organized the data collection process, equally contributed to data analysis, drafting or revising the article, gave final approval of the version to be published, and agree to be accountable for all aspects of the work.

## Funding

This study was financially supported by Haramaya University (Grant No. HUCF-2020-02-NA-06).

## Conflict of Interest

The authors declare that the research was conducted in the absence of any commercial or financial relationships that could be construed as a potential conflict of interest.

## Publisher's Note

All claims expressed in this article are solely those of the authors and do not necessarily represent those of their affiliated organizations, or those of the publisher, the editors and the reviewers. Any product that may be evaluated in this article, or claim that may be made by its manufacturer, is not guaranteed or endorsed by the publisher.

## Abbreviations

AOR: adjusted odds ratio
ASSIST: Alcohol Smoking and Substance Involvement Screening Test
CI: Confidence Interval
COR: Crude Odds Ratio
COVID-19: Corona Virus Disease 2019
SRQ-20: Self Reporting Questionnaire.
